# Successful induction of pseudopregnancy using sonic vibration in mice

**DOI:** 10.1038/s41598-023-30774-x

**Published:** 2023-03-03

**Authors:** Yui Wake, Marina Endo, Shigemi Tsunoda, Hirosuke Tawara, Hisayuki Abe, Yuki Nakagawa, Takehito Kaneko

**Affiliations:** 1 Division of Science and Engineering, Graduate School of Arts and Science, Iwate University, Iwate, 020-8551 Japan; 2 Institute for Animal Reproduction, Ibaraki, 300-0134 Japan; 3 Department of Chemistry and Biological Sciences, Faculty of Science and Engineering, Iwate University, Iwate, 020-8551 Japan

**Keywords:** Animal biotechnology, Cell biology

## Abstract

Embryo transfer (ET) is an essential reproductive technology for the production of new animal strains and maintenance of genetic resources. We developed a method, named Easy-ET, to induce pseudopregnancy in female rats by artificial stimulation using sonic vibration instead of mating with vasectomized males. This study examined the application of this method for the induction of pseudopregnancy in mice. Offspring were obtained from two-cell embryos transferred into females with pseudopregnancy induced using sonic vibration in proestrus on the day before embryo transfer. Furthermore, high developmental rates of offspring were observed when pronuclear and two-cell embryos were transferred to females in estrus that were stimulated on the day of embryo transfer. Genome-edited mice were also obtained using frozen-warmed pronuclear embryos with clustered regularly interspaced short palindromic repeat (CRISPR)/CRISPR-associated system (Cas) nucleases introduced using the technique for animal knockout system by electroporation (TAKE) method, which were transferred to females with pseudopregnancy induced on the day of embryo transfer. This study demonstrated that induction of pseudopregnancy by sonic vibration was also possible in mice.

## Introduction

Reproductive technologies are essential for producing new animal strains and maintaining genetic resources^1–3^. Embryo transfer (ET), a reproductive technology, is routinely used in laboratory and domestic animals^4–11^. The use of ET has recently increased owing to the production of many types of genetically modified laboratory animals for the study of human diseases^12,13^. Genome editing technology has further accelerated the frequency of the production of genetically modified strains^14–16^. Various human disease model strains can be produced by introducing nucleases via the CRISPR-Cas system into embryos using the microinjection^17^ and electroporation method (TAKE—technique for animal knockout system by electroporation)^18–20^. ET is required for the efficient production of these strains and regeneration of valuable strains from frozen embryos^21–23^ and freeze-dried sperm^24–26^ that are preserved as genetic resources.

In mice and rats, females require mating stimulation for the maintenance of pregnancy. The induction of pseudopregnancy is required to transfer embryos produced under in vitro conditions into the oviducts of females. Pseudopregnancy for ET is typically induced by mating with vasectomized males overnight on the day before ET is performed. However, this procedure requires a large breeding space, and it is costly to maintain a sufficient number of healthy females and vasectomized males to induce pseudopregnancy. We recently developed method, named Easy-ET, to induce pseudopregnancy in female rats by artificial stimulation using sonic vibration instead of vasectomized males^27^. The production of female mice with pseudopregnancy induced using sonic vibration was as efficient as rats. This study examined the application of sonic vibration to induce pseudopregnancy in mice.

## Results

Table 1 shows the development of embryos transferred to females with pseudopregnancy induced via sonic vibration. Artificial stimulation by sonic vibration was performed with females in proestrus seven times with 30 s per stimulation on the day before embryo transfer, according to our procedure in rats^27^. Of all two-cell embryos transferred to these females with pseudopregnancy, 21.8% were implanted, and 12.7% of embryos developed into offspring. In the control group comprising females with pseudopregnancy induced by mating with vasectomized males, 61.0% of transferred embryos were implanted, and 34.1% developed into offspring. Embryo development using artificially stimulated females was significantly lower than that of the control group in which pseudopregnancy was induced in females by mating with vasectomized males.

**Table 1:** Development of embryos transferred to females with pseudopregnancy induced by sonic vibration.

Pseudopregnancy	Time of stimulation	Embryos	No. of embryos transferred	No. of females (No. of females pregnant)	No. (% ± SD) [litter size] of embryos implanted	No. (% ± SD) [litter size] of offspring
With vasectomized male	–	Two-cell	41	3 (3)	25 (61.0 ± 4.3) [5–11]^a^	14 (34.1 ± 8.4) [3–7]^e^
Sonic vibration	Day before embryo transfer	Two-cell	55	4 (3)	12 (21.8 ± 8.3) [0–6]^b^	7 (12.7 ± 4.6) [0–3]^f^
	Day of embryo transfer	Two-cell	65	4 (4)	29 (44.6 ± 8.1) [4–11]^c^	27 (41.5 ± 9.5) [3–11]^g^
	Day of embryo transfer	Pronuclear	61	4 (4)	44 (72.1 ± 5.8) [9–13]^d^	42 (68.9 ± 8.4) [7–13]^h^

Significant differences at *P* < 0.05: a versus b, b versus c and d, c versus d, e versus f, f versus g and h, g versus h.

In this study, estrus females were stimulated on the day of embryo transfer. Of all two-cell embryos transferred to these females with pseudopregnancy, 44.6% were implanted, and 41.5% of embryos developed into offspring. Furthermore, when pronuclear embryos were transferred, 72.1% were implanted, and 68.9% of embryos developed into offspring. No significant differences were observed in the development of transferred two-cell and pronuclear embryos compared to those in the control. High embryo development was observed when pronuclear embryos were transferred into females in estrus and stimulated on the day of embryo transfer.

This study examined the production of genome-edited mice using an artificial pseudopregnancy procedure. Frozen-warmed pronuclear embryos that were introduced with tyrosinase-targeting guide RNA and Cas9 protein using the TAKE method were transferred to females in estrus, which were stimulated on the day of embryo transfer. Of all embryos transferred into these females with pseudopregnancy, 28.3% were implanted, and 24.5% of embryos developed offspring. A total of 38.5% of offspring showed knockout of the targeted gene (Table 2).

**Table 2:** Development of frozen-warmed embryos with genome editing performed using the TAKE method, which were then transferred to females with pseudopregnancy induced by sonic vibration on the day of embryo transfer.

No. of embryos transferred	No. of females (No. of females pregnant)	No. (% ± SD) [litter size] of embryos implanted	No. (% ± SD) [litter size] of offspring	No. (% ± SD) [litter size] KO offspring
53	3 (3)	15 (28.3 ± 4.4) [4–6]	13 (24.5 ± 0.5) [4, 5]	5 (38.5 ± 7.0) [0–4]

*KO* knockout, *TAKE* Technique for Animal Knockout System by Electroporation.

## Discussion

We recently developed the method, named Easy-ET, to induce pseudopregnancy in female rats by artificial stimulation using sonic vibration instead of mating with vasectomized males^27^. This study examined the application of sonic vibration to induce pseudopregnancy in mice. The results showed that induction of pseudopregnancy by sonic vibration was also possible in mice, similar to that in rats. The induction of pseudopregnancy by vaginal cervical vibration has been previously reported^28,29^. In these reports, successful induction of pseudopregnancy was evaluated by observation of the uterine condition after stimulation. Our results further demonstrated the abilities of embryo implantation and continuation of pregnancy by stimulation using sonic vibration.

Normal offspring were obtained from two-cell embryos transferred to females in proestrus on the day before embryo transfer. Although these results indicate that pseudopregnancy could also be induced in female mice by artificial stimulation using sonic vibration, the developmental rate of offspring was lower than that of the control group, in which pseudopregnancy was induced in females via mating with vasectomized males. Pseudopregnancy by sonic vibration was induced earlier than that induced by mating with vasectomized males because females usually mate with males overnight. However, no significant differences were observed in the development of offspring of two-cell embryos transferred to females in estrus stimulated on the day of embryo transfer compared to those in the control. This stimulation process was conducted later than that performed with vasectomized males. The detailed mechanism underlying the induction of pseudopregnancy by sonic vibration in this study remains unknown. Further studies concerning endocrine and biochemical analyses are required for the efficient induction of pseudopregnancy and the establishment of an optimum protocol.

In addition, high embryo development was observed when pronuclear embryos were transferred to females in estrus when stimulated on the day of embryo transfer. Furthermore, genome-edited mice were obtained using frozen-warmed pronuclear embryos with nucleases introduced using the TAKE method, which were transferred to females in estrus stimulated on the day of embryo transfer. Positive results were observed with this one-day operation, comprising pseudopregnancy, genome editing of embryos, and ET. Recently, many genome-edited animals have been produced using the CRISPR-Cas9 system. A one-day operation involving the transfer of frozen embryos^21–23^, performance of the TAKE method^18–20^, induction of pseudopregnancy by sonic vibration, and ET can contribute to the production of genome-edited animals.

Previously, we successfully induced pseudopregnancy using sonic vibration in rats. This study demonstrated that this method could be applied to mice. This method can contribute to the production of new strains, maintenance of genetic resources, and the removal of pathogens. Furthermore, females with pseudopregnancy are generally produced by mating with vasectomized males. Maintenance of a sufficient number of healthy females and vasectomized males requires a large space and is expensive. Artificial pseudopregnancy induced using sonic vibration did not require maintenance of vasectomized males. The procedure is economical as it does not involve large breeding spaces and high expenses for inducing pseudopregnancy and contributes to animal welfare and follows the 3Rs of appropriate animal handling in laboratories by minimizing the use of laboratory animals needed for performing it.

## Methods

### Animals

Crlj:ICR mice (Charles River Laboratories Japan Inc., Yokohama, Japan) were used for embryo collection and subsequent transfer. C57BL/6 J mice (Charles River Laboratories Japan Inc.) were used for the genome editing of embryos. The animals were maintained in plastic cages in an air-conditioned (temperature 23 °C ± 3 °C, humidity 50% ± 10%) and light-controlled room (illuminated from 07:00 to 19:00 h). All animal care and procedures performed in this study were reported in accordance with ARRIVE guidelines, and were approved by the Animal Research Committee of Iwate University and the Institute for Animal Reproduction. All methods were carried out in accordance with relevant guidelines and regulations.

### Embryo collection

Crlj:ICR females aged 9–13 weeks were induced superovulation by intraperitoneal injection of 10 IU/body pregnant mare serum gonadotropin (PMSG; ASKA Animal Health Co., Ltd., Tokyo, Japan), followed by intraperitoneal injection of 10 IU/body human chorionic gonadotropin (hCG; ASKA Animal Health Co., Ltd.) 48 h later. These females were then mated overnight with Crlj:ICR males aged > 10 weeks. The presence of vaginal plugs in the females confirmed that mating had occurred. Pronuclear stage embryos were collected by flushing the oviducts with PB1 medium^30^ on the day after the mating. These embryos were then cultured to a two-cell stage in a fresh KSOM medium^31^ at 37 °C in an atmosphere containing 5% CO_2_ and 95% air. Pronuclear embryos produced from Crlj:ICR females and C57BL/6 J males were vitrified for subsequent genome editing.

### Genome editing of embryos

Genome editing of frozen-warmed embryos was performed using the TAKE method^18–20^. A super-electroporator NEPA21 (NEPA GENE Co. Ltd., Chiba, Japan) was used to introduce nucleases. The nuclease solution comprised 100 μg/mL of Cas9 protein (Integrated DNA Technologies Inc. Coralville, IA, USA) and 500 μg/mL of dual RNA (mixture of crRNA and tracrRNA, Integrated DNA Technologies Inc.) in Opti-MEM (Thermo Fisher Scientific Inc., MA, USA). crRNA was designed to target the tyrosinase gene in C57BL/6 mice (5′-GGGTGGATGACCGTGAGTCC-3′)^32^. The nuclease solution (5 μl) was placed between metal plates of electrodes with a gap of 1 mm on a glass slide (CUY501P1-1.5, NEPA GENE Co. Ltd.). Embryos were placed in a line between electrodes. The poring pulse was set to voltage: 40 V, pulse length: 2.0 ms, pulse interval: 50 ms, number of pulses: 4, decay rate: 10%, polarity: + . The transfer pulse was set to a voltage: 15 V, pulse length: 50 ms, pulse interval: 50 ms, number of pulses: 5, decay rate: 40%, polarity: + / − . The genome editing rates of the offspring were estimated using the eye color difference (white: successful genome edition; black: not successful).

### Artificial pseudopregnancy of females

**Figure 1:**
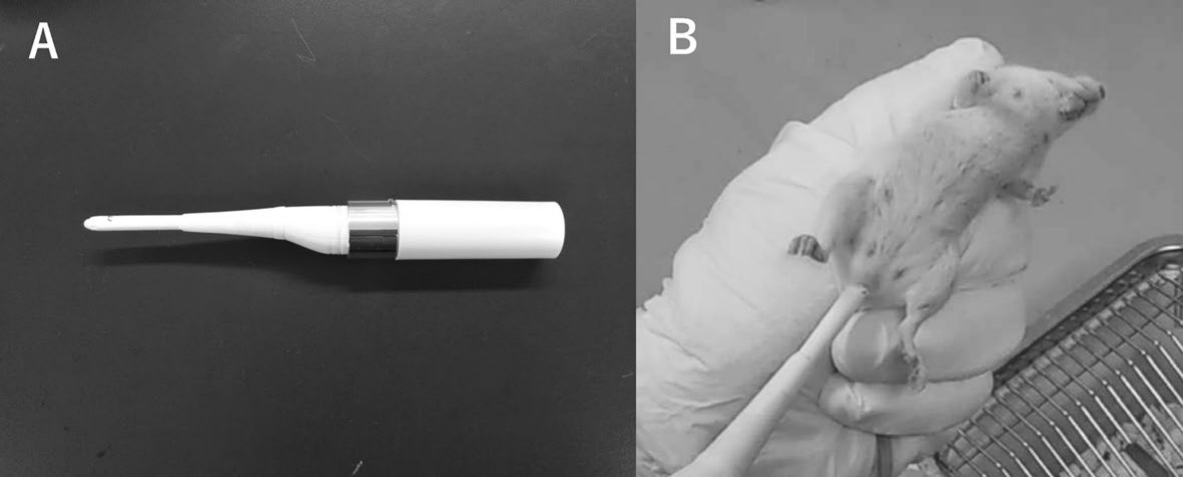
Sonic vibrator (**A**). Probe was inserted in the vagina of female (**B**).

Induction of pseudopregnancy was performed using a previously published method in rats^27^. A self-made sonic vibrator (probe diameter: 5 mm, length: 4 cm) was used for the artificial stimulation (Fig. 1A). The probe of the vibrator was inserted into the vagina of the female aged 8–13 weeks and stimulated seven times at 30 s intervals with 30 s per stimulation (Fig. 1B). The estrus cycle of females was decided by observation of external genitalia. Stimulation was performed in females in proestrus at 16:00 on the day before embryo transfer and in estrus at 9:00 on the day of embryo transfer. In the control group, pseudopregnancy was induced by mating with vasectomized males.

### Embryo transfer

The two-cell embryos were transferred into the oviducts of females with pseudopregnancy that were stimulated the day before embryo transfer or mated with vasectomized males. Pronuclear, two-cell, or genome-edited pronuclear embryos were transferred into the oviducts of females with pseudopregnancy that were stimulated on the day of embryo transfer. Females were anesthetized using the mixture of medetomidine, midazolam, and butorphanol during operation. The number of implantation sites and offspring were counted after euthanasia by cervical dislocation at 18 days following gestation.

### Data analysis

Data were analyzed using the chi-square test followed by a multiple comparison test using Ryan’s method.

## Data Availability

The authors declare that the data supporting the findings of this study are available within the paper.
